# Compulsory notification of prion diseases in Brazil: What has changed since 2005?

**DOI:** 10.1590/S1980-57642009DN20200014

**Published:** 2008

**Authors:** José Eriton Gomes da Cunha, João Ricardo M. Oliveira

**Affiliations:** 1 Biological Sciences Center –Undergraduate program- Federal University of Pernambuco (UFPE).; 2 Department of Neuropsychiatry and Keizo Asami Laboratory (LIKA) - Federal University of Pernambuco (UFPE), Recife – Pernambuco, Brazil.

## To the Editor

Recently, in an article published by Dementia & Neuropsychology, Martins et al. (2007)^1^ screened mutations and polymorphisms at the prion gene (PRNP) in 35 cases of Creutzfeldt-Jakob disease (CJD) from 13 Brazilian states, reported to the Health Surveillance Secretariat/Ministry of Health (SVS/MS), between 2005 and 2007. This study is highly important because prion diseases (*PD*) are linked to mutations in the PRNP while some polymorphisms at the same gene are considered risk factors for these groups of disorders. Numbering among them is the polymorphism of the codon 129 which has been described in other articles as the most significant risk factor for such disorders.^2,3^

After thoughtful reading we would like to discuss some points that we feel should be addressed. One of these is a minor misunderstanding in the *Abstract and Results* sections of the main text. Regarding the first, the authors mentioned that “twenty seven cases (74%) were classified as possible sCJD while 51% were classified as probable sCJD”. However, these 74% (which corresponds to 26 and not 27 cases as stated in the Article) represent the total of sCJD (possible + probable). In *Results*, the statement “sCJD possible (probable + possible)” is inadequate, because it suggests that cases of “possible sCJD” is the sum of possible and probable cases. However, these small mistakes do not detract from the importance of this work.

Analyzing the epidemiological data, we observed that only 35 cases reported in two years by SVS/MS included complementary examinations of blood and / or cerebral spinal fluid (CSF). Three of these were studied with Neuropathologic analysis, but only two confirmed the presence of prion protein aggregate. These procedures are crucial to exclude other etiologies and to better analyze these disorders which often presenting with clinical heterogeneity.

The data gathered so far by Martins et al. are highly relevant and promising, showing that 89% of the compulsory notification forms were filled out properly, enriching epidemiological analysis of this neglected disorder.

Most of the biological samples examined to confirm this diagnosis were sent to reference centers in São Paulo, showing that only one Brazilian state is leading this process. However, we should have at least one reference center per region, considering the continental dimensions of Brazil. This is especially true if we consider the potential consequences to the Brazilian Health system and Economy in the event of a possible epidemic.

Last December, at the VI RPDA (Reunião de Pesquisadores em Doença de Alzheimer e Desordens Correlacionadas)^4^ we reported that, up until 2004, the epidemiology about CJD was not reliable because there were many communication problems between the state secretariats of health and the Health Ministry. Nevertheless, in 2005, the Health Ministry included PD in the compulsory notification list, which requires health care professionals to notify all suspected cases to public departments of health in order to establish groups of epidemiological surveillance, reinforcing the PD epidemiology.^5^

Between 2006 and 2007, we visited the state secretariats of health in Minas Gerais (MG), Pernambuco (PE), Rio Grande do Norte (RN) and São Paulo (SP) and noted that only São Paulo presented an active and organized group of epidemiological surveillance for *PD*, adopting what is considered standard model.

After the visits, we maintained contact with the RN and PE secretariats, but so far the scenario is the same.

Currently, the Secretariat of Health in Pernambuco is concerned about forming a group of epidemiological surveillance for these kinds of diseases. A lecture was held last February with the presence of various members of the State Health technical staff to increase awareness for technical management of *PD*.

After 3 years of effort to form a network of epidemiological surveillance for *PD*, the public health services still face several challenges including lack of personnel able to identify clinical and laboratorial aspects involved with *PD*, and scant resources to supply the necessary technical procedures to increase diagnosis accuracy, besides the centralization of the study in São Paulo. The sum of all these factors compromise the setting up of PD study centers across the country.

The study by Martins et al., reviewed in this article, reflects the dedication of the Ludwig Institute for Cancer Research in São Paulo, especially by Dr Vilma Martins, during the last ten years studying the prion protein and its related diseases, which are improperly studied in some countries including Brazil, due to the obstacles previously described.
