# Supplementary material for: Compulsory notification of prion diseases in Brazil: What has changed since 2005?
Source: Dement Neuropsychol. 2008 Apr-Jun;2(2):155–6. doi: 10.1590/S1980-57642009DN20200014 (PMC5619587; doi:10.1590/S1980-57642009DN20200014)
Supplement: Related texts

# Reply from the authors

**Authors:** Vilma Regina Martins, Hélio Rodrigues Gomes, Leila Chimelli, Sergio Rosemberg, Michele Christine Landemberger

Regarding the comments from Gomes da Cunha & Oliveira we acknowledge the error in the Abstract of our manuscript where a total of twenty six (as described in the *results*) instead of twenty seven cases were classified as possible sCJD. In their letter the authors also mentioned our statement in the *results* section that possible CJD may represent the sum of possible and probable sCJD. In fact, in that specific paragraph we were describing Table 1. From the total cases of notified patients 74% were initially classified, based on clinical parameters, as possible sCJD. When these patients were analyzed for positive levels of 14.3.3, 51% or the total were confirmed as probable sCJD while 23% did not fulfill criteria for this classification and continued to be classified as possible sCJD. Indeed, in this specific case (Table 1) the group representing 74% of the patients initially classified as possible sCJD, contains patients that were later classified as probable CJD (51%) where 23% of the patients remained classified as possible sCJD.

We agree with Gomes da Cunha & Oliveira that there is a need for improved communication between some State Secretariats and the Ministry of Heath. However, we have handled notified cases, and had samples submitted for biochemical analysis, from all over the country indicating that a working minimal framework is in place. Nevertheless, there is a special need to make an effective statement on compulsory notification of these diseases to the medical community, particularly to neurologists and psychiatrics. Indeed, proper notification of suspected cases is the first step toward establishing the epidemiological surveillance of prion diseases in Brazil.

On the other hand, we disagree with Gomes da Cunha & Oliveira that reference centers for diagnosis must be created in each Brazilian region. We know that the incidence of these diseases is about 1 in 1,000,000 inhabitants/year. Given the IBGE (Brazilian Institute of Geography and Statistics) have estimated that the Brazilian population in 2004 was around 180,000,000, we expected to have 180 to 200 cases of CJD a year in Brazil. This is a very low number of patients to justify investment in acquiring equipments and training personnel in different regions. Costs will be much lower if the diagnosis is concentrated at one reference center, independently of where it is located in Brazil. This is exactly the approach adopted in other counties such as Argentina, USA, Canada, England and Germany.

Indeed, we have to concentrate effort in each State to identify, notify and follow all the possible cases of prion disease in Brazil. The adequate epidemiological surveillance of patients with prion diseases shall allow Brazil to contribute to clinical trials developing drugs to treat these devastating diseases.
